# O015: A novel antibacterial material for transparent dressings

**DOI:** 10.1186/2047-2994-2-S1-O15

**Published:** 2013-06-20

**Authors:** W Zingg, L Clack, C Ginet, H Ney, G Renzi, M Black, M Martin

**Affiliations:** 1 Infection Control Program, University of Geneva Hospitals, Geneva, Switzerland; 2 Department of Sterilisation, University of Geneva Hospitals, Geneva, Switzerland; 3 Molecular Biology Lab, University of Geneva Hospitals, Geneva, Switzerland; 4 Palm Beach Children's Hospital, Palm Beach, USA; 5 Infection control program, Regionale Gesundheitsholding Heilbronn-Franken, Helibronn, Germany

## Introduction

Intravascular lines are indispensable in hospital care. The main complication of their use is catheter-related bloodstream infection (CRBSI). Transparent, semipermeable dressings (TSD)are standard in covering the insertion site. ‘Bionate’ is a polymer with antibacterial properties and yet has not been taken up by a company to be marketed as a medical product. The polymer backbone consists of polyurethane while the surface consists of self-assembling monolayer end groups of quaternary ammonium structures. These structure properties make the material a candidate to produce easy-to-use antibacterial TSD.

## Objectives

The aim of the study was to assess the effectiveness of the material as a growth inhibitor of microorganisms on the skin of healthy volunteers.

## Methods

Sterile ‘Bionate’ and control patches were applied to undisinfected skin (upper arm) of 10 healthy volunteers for 3, 5, and 7 days. Five volunteers tested the patch for 10 days. After removal, a sterile, moistened cotton swab was taken from the skin site and the patches were put into normal sterile saline. Skin swabs were transferred into 3ml sterile saline and incubated for 2 minutes at room temperature. Patches in saline were vortexed and incubated for at least 15 minutes at room temperature. Dilution series were prepared and 100ul of each probe were put onto trypticase-soy-agar for growth and colony counts. Agar plates were incubated at 35°C for up to 48h and colony forming units (CFU) were counted thereafter.

## Results

Log growth differences of skin swabs between ‘Bionate’ and control patches on days 3, 5, 7, and 10 were 1.4, 2.5, 2.0, and 1.4, respectively. Growth difference was significant from day 7 onwards (P=0.002). Log growth differences of the ‘Bionate’ and control patches on days 3, 5, 7, and 10 were 1.8, 1.8, 2.5, and 1.9, respectively. Growth reduction was significant from day 5 onwards (P=0.015).

## Conclusion

The study identified remarkable growth of microorganisms on skin covered with polyurethane dressings. Growth reduction of ‘Bionate’ as compared to standard polyurethane makes this material promising to develop a future TSD with inherent antimicrobial properties.

## Disclosure of interest

None declared.

